# The recent development and applications of fluidic channels by 3D printing

**DOI:** 10.1186/s12929-017-0384-2

**Published:** 2017-10-18

**Authors:** Yufeng Zhou

**Affiliations:** Singapore Centre for 3D Printing (SC3DP), School of Mechanical and Aerospace Engineering, Nanyang Technological University, 50 Nanyang Ave, Singapore, 639798 Singapore

**Keywords:** Fluidic channel, Lab-on-a-chip, 3D printing, Diagnosis, Tissue engineering, Reactionware

## Abstract

The technology of “Lab-on-a-Chip” allows the synthesis and analysis of chemicals and biological substance within a portable or handheld device. The 3D printed structures enable precise control of various geometries. The combination of these two technologies in recent years makes a significant progress. The current approaches of 3D printing, such as stereolithography, polyjet, and fused deposition modeling, are introduced. Their manufacture specifications, such as surface roughness, resolution, replication fidelity, cost, and fabrication time, are compared with each other. Finally, novel application of 3D printed channel in biology are reviewed, including pathogenic bacteria detection using magnetic nanoparticle clusters in a helical microchannel, cell stimulation by 3D chemical gradients, perfused functional vascular channels, 3D tissue construct, organ-on-a-chip, and miniaturized fluidic “reactionware” devices for chemical syntheses. Overall, the 3D printed fluidic chip is becoming a powerful tool in the both medical and chemical industries.

## Background

Shrinking the bulky and costly laboratory equipment into a single small, user-friendly, easily replicable chip provides a significant advantage over traditional assays. Lab-on-a-chip (LOC) technologies or micro-total analysis systems (μTAS) with microfluidics have been continuously evolving from simple single-function devices to analytical systems with multiple functionalities and revolutionizing the research fields of chemistry, physics, pharmacology, cell biology, chemical biology, neuroscience, biomechanics, bioanalysis, and tissue engineering [1–5]. Particularly, they have ubiquitous presences in various clinical and forensic analysis [6], such as cell sorting and isolation [7, 8], cellular analysis [9], biosensor and point-of-care (POC) diagnosis [9, 10], pharmacological screening [2, 11, 12], proteomics and metabolomics [9], immunoassays [13], genetic analysis or genomics [14], multi-cellular tissue spheroid fabrication, organ-on-a-chip using different tissue spheroids [15–20], and bioreactor for co-culture and maturation of micro-organs. Current microfluidic systems have extended their applications with the integration of several functions, for example, cell/tissue incubation, enzymatic processing, biochemical analysis, optoelectronic measurement, and computer-controlled microfluidics. In comparison to the traditional macro-scale methods, these microfluidic chips have the capabilities of (1) streamlining complex assay protocols, (2) minimizing the sample and reagent volumes, (3) maximizing the measurement of precious sample at the reduce performance time, power consumption, and substantial cost, (4) accurately manipulating the cell microenvironment, and (5) providing scalability and batch screening of multiple samples in a massive parallel style. Despite the rapid development of microfluidics and applications in biological research and biomedical engineering over the past decades, the wide and practical acceptance (e.g., commercial point-of-care testing) is still not very satisfactory. One of the reasons may be the requirement of a highly adaptable, rapid, and easy process of fabricating the microfluidic systems with increasing prevalence and complexity and the absence of a “killer application” that would outperform existing traditional methods.

3D microfluidic chips can overcome the limitations of conventional 2D designs and have the potential advantages of improved observation efficiency [21], continuous 3D motion [22], and integration of more functions, especially for patterning with liquids. With the increases in the complexity and more sophisticated tasks, the transport of different fluid streams becomes easy in 3D configuration. However, the complexity of manufacturing such systems (e.g., more steps for pattern fabrication, alignment, and sealing in photolithography in comparison to the 2D configuration) has deterred their wide use. There are several approaches for mass replication and production of 3D microfluidic devices, for example, micro-machining, casting, hot embossing, in situ construction injection, and laser ablation [23]. Some of them require large equipment space, intensive labor, time consumption and suffer from limited bio-compatible materials. It is troublesome to produce multiple photomasks in high resolution (<10 μm) and align and expose sequential layers of photoresist for the soft lithography. The need for fabricating the replication master limits its application on a smaller scale, for example, academic research and biological applications. In addition, the equipment complexity and operator’s skills are relatively high. Therefore, a quick, easy, and direct fabrication is preferred for the end users. Recently, advances in 3D printing may simplify the fabrication process of fluidic devices into a single step [24–26].

Additive manufacturing or 3D printing could produce complicated intricate architectures effectively at relatively low cost and infrastructure investment, but high attainability. Freedom of product design for manufacturing and assembly greatly encourages product innovation universally available to any customers by creating unique bespoke one-off objects. This technology has witnessed an explosive growth in the manufacturing industry and the consumer market. Because no etching or dissolution is required, the process of adding materials is environmentally friendly and economically efficient. The global market for 3D printing has increased to $4.1 billion in 2014 and may reach $20.2 billion by 2020 [13]. Although 3D printed structures cannot compete with those manufactured by photolithography in the resolution now, they enable the enhanced geometrical control of the unprecedented channel shape and complexity (e.g., cross-section and height) quickly and inexpensively that has been previously impossible. In comparison to the conventional clean-room-based photolithography, it offers several major advantages: (1) rapid prototyping and replication of products with its attendant benefits and full automation to positively disrupt the development cycles, (2) greatly simplifying the manufacturing process without a replication master, assembly, and extensive labor, (3) no requirement of clean-room environment for comparatively low cost in manufacturing infrastructure, (4) arbitrary channel shapes instead of rectangular one in photolithography, (5) very simple procedures for structures with various heights in a single step instead of using the layer-by-layer strategy, (6) molding suspended structures without any alignment or sacrificial parts, (7) multiple materials for various applications (e.g., artificial tissue scaffolds), (8) dramatically lowering the barrier to creating sophisticated 3D biomedical models, (9) the great topography flexibility of multiple 2D layers stacked together, and (10) enabling users to adopt a “fail fast and often” strategy [27].

In this paper, the recent advances in 3D printing in the fluidic channel and its applications in biology and biomedical engineering are reviewed. First, all materials used in the fabrication are summarized. Then, various manufacturing approaches, such as stereolithography (SL), two photon polymerization (2PP), fused deposition modeling (FDM), polyjet, and 3D bioprinting, are introduced. The strategy of removing the scaffold made by 3D printing also enables the fabrication of channels in high complexities and throughput. Currently investigated applications, such as the deposition and detection of cells and proteins, development of bacterial communities, formation of the microvascular network, stimulation of cell growth, and construction of 3D tissue/organ, are listed. Finally, the trend of 3D printing and fluidic channel in the lab-on-a-chip is discussed, and the improvement on the current limitations is necessary for the fast commercialization and wide acceptance of “killer-applications”.

## Materials

Materials used for the fabrication of a microfluidic system require the consideration of their function, the degree of integration, and biological application, for example, cellular compatibility, supportability (e.g., oxygen and nutrient diffusion), optical transparency, and mechanical properties. The most popular materials in molding approaches are polydimethylsiloxane (PDMS) and thermoplastics. Devices molded in thermoplastics [e.g., polystyrene, polymethyl methacrylate (PMMA), polyurethane] enable higher throughput but do not necessarily allow superior manufacturability. However, thermoplastics and injection molding are not amenable to rapid-prototyping because both the equipment and the molds are expensive, the turn-around times for the fabrication of metallic molds can be on the order of weeks, and the molding procedure requires substantial expertise. Plastics do not have the high gas solubility as PDMS which obeys Henry’s law.

PDMS is usually selected because of its (1) gas permeability for keeping cells and bacteria alive for a long time, (2) elasticity (Young’s modulus of 2 MPa) [28], capable of making micro-pumps and valves approximately 1000 times smaller than that of hard plastics, (3) simple chemical modification using well-known silane chemistry, (4) optical transparency at the wavelength of 240–1100 nm [29], nontoxic, electrically insulating, and impermeability to liquids, (5) conformal and easy to mold with high fidelity and precision (in the order of 10 nm) [29], (6) fairly low cost, (7) free copyright, (8) biocompatible, and (9) rapid prototype using simple procedures. However, with an increasing focus on translation and low-cost devices, molding approaches illustrate their shortcomings: (1) PDMS molding (curing, assembly, bonding, and inlet punching) using photolithography is substantially labor-intensive and complex for inexperienced people [30] so that it is hard to fully automate and disseminate out of research labs for commercialization or large-scale production [31]; (2) the user interfaces (inlets/outlets) of PDMS chips consisted of punched or molded holes are prone to leakage and awkward to connect in comparison to the leak-free connectors (e.g., Luer-lock, barbed connectors); (3) engineering expertise and equipment (e.g., computer, pressure sources, software) required for the operation of fluidic valves and connection of chips are absent in most biomedical laboratories; (4) multiple layers of PDMS must be fabricated by standard methods that is tediously slow and then sealed together for a 3D channel (multilevel channels or a single channel with different sizes), which limits the complexity of 3D constructs; (5) PDMS is a very porous matrix that swells in organic solvents, resulting in the loss of solvent into the microchannel walls, detachment of the seal between the channels and the surface, and alterations of the channel geometries.

Because PDMS is unable to be directly printed, other materials are also employed. Biocompatible and transparent resins provide the possibility of fabricating biomedical devices by stereolithography (SL) although most of SL resins are non-biocompatible and translucent or opaque materials in the jewelry and structural modeling. Other properties in choosing the resin for SL-fabricated fluidic devices are gas permeability, hydrophobicity, and chemical stability in the presence of solvents. The currently popular biostable resins are based on polyester/polyether oligomers with acrylate or methacrylate functions and biodegradable composites of methacrylate-functionalized polyesters. Fibers of polyethylene and nylon have proven to be an excellent choice for preparation of 3D elements. Heating the polymer wires above their glass-transition temperatures but below their melting points allows for forming the desired shapes.

WaterShed is nearly colorless with a clarity, flexibility, and hardness similar to polycarbonate or poly(methyl methacrylate). Furthermore, it does not swell in water and meets biocompatibility standards ISO 10993–5 (cytotoxicity), ISO 10993–10 (sensitization), ISO 10993–10 (irritation), and USP Class VI [32]. However, longer-term cytocompatibility of WaterShed needs further investigation [33]. Internal processing of PMMA, PDMS, polystyrene (PS), and polyvinyl alcohol (PVA) polymers has also been investigated. Polypropylene (PP) is an attractive material for the fabrication of micro- and milli-reactionware as it is a robust, flexible, and chemically inert polymer, and significantly less expensive than PDMS.

Naturally derived polymer (e.g., alginate, gelatin, collagen, fibrinogen, agarose, chitosan, fibrin, and hyaluronic acid) or modified proteins (gelatin methacrylate) isolated from animal or human tissue for 3D bioprinting has the similar property to the human extracellular matrix (ECM) and inherent bioactivity [34]. Meanwhile, synthetic polymers and molecules [e.g., polyethylene glycol (PEG), PEG amine] can be tailored to specific physical properties for specific applications but has poor biocompatibility, toxicity, and loss of mechanical properties during degradation [35]. Synthetic hydrogels are both hydrophilic and absorbent, especially attractive for regenerative medicine [36]. Synthetic–natural mixtures are also used to combine their advantages. In the 3D bio-printing of vascularized tissue constructs, the preparation of bioink composed of cells suspended in a liquid pre-gel solution is critical [37]. During the printing process using mechanical extrusion, the bioink is gelled by polymer crosslinkers, photo activation, or thermal activation to form a hydrogel that physically constrains the homogeneously suspended intact cells without compromising the cell viability and organelle activity illustrated by fluorescent assays and organelle tracking even after 48 h of culture, which is due to the similar mechanical characteristics of 3D crosslinked hydrophilic polymer networks in the hydrogels to that of ECM [38]. By varying the concentration of crosslink, the hydrogel can be tuned to be “soft” or “robust” gels [38]. The cellular proliferation should be high to populate the printed construct but be maintained at an appropriate rate to achieve tissue homeostasis without hyperplasia. The DNA bioink is advantageous over synthetic polymer hydrogel because of its higher biodegradability.

## Manufacture

### Stereolithography (SL)

Stereolithography is the most popular 3D printing approach to directly print the micro-channels or create modular structures. As opposed to molding processes, SL is fully digital, amenable to finite element modeling (FEM), intrinsically modular, and able to simplify the commercialization pathway [39]. The single-photon polymerization (1PP) process occurs near the surface of a photosensitive resin. The outcome of SL is dependent upon the laser spot size and the absorption spectra of the photo-resins. It presents an inherent advantage in the production of 3D structures over other lithographic methods (e.g., photolithography and soft lithography) owing to no need of alignment or bonding. Laser raster scanning, laser vector scanning, and digital light processing (DLP) have been developed for curing the resins in commercial SL instruments [40]. In DLP-SL, an entire layer of resin is exposed at once so that its resolution is determined by the projected pixel size. Digital micromirror display (DMD) technology and commercially available projectors allow reducing the price of DLP-SL printers significantly (e.g., ~$100). The structural fidelity is superior in the free surface technique over the constrained one because the mechanical separation in the bat configuration can induce stress fractures or bend of delicate features and increase roughness between layers. However, the resin reservoir depth limits the object height in the free surface technique, but not in the bat configuration. Furthermore, the curing time is shorter in the bat configuration, where the photo-polymerization inhibited by oxygen occurs away from the air-resin interface. The achievable layer thickness is only dependent on the Z stage resolution, but not the resin viscosity. The large discrepancy in the price of SL printers is attributed to resolution, build area and speed. Printed reusable templates have resolutions of 50 μm and up to 10 μm in localized hindrances, and can be fabricated within 20 min at an average cost of $0.48 [41]. The main limiting factors of SL are the effective drainage of the uncured liquid resin, optical clarity, and Z-height resolution. SL printers cannot change the printing materials easily, but both resolution and surface finish are sufficient to make PDMS templates with the combination of thick and thin features.

In two-photon polymerization (2PP), two photons from femtosecond pulsed near-infrared lasers are absorbed simultaneously by the photo-initiator, directly recording or writing an arbitrary polymeric 3D pattern into a volume of photosensitive material. 2PP is not limited by the laser diffraction, resulting in much higher resolution (e.g., ~100 nm) in comparison to 1PP. For the negative photoresists such as those containing acrylic oligomers or epoxy resins, 2PP produces the crosslinking of polymer chains through radical polymerization and makes the exposed area insoluble in the solvent, which provides the possibility of directly writing the structure. For the positive photoresists, 2PP causes the polymeric chains to break and become soluble in the solvent to write the reverse structure. Although commercial negative photoresists have better capacities of modeling and conformity, they are not commonly used for fabricating fluidic chips because of the long processing time. Femtosecond lasers induce a local phase change in the photo-sensitive glass (e.g., Foturan) from amorphous to crystalline and can produce sub-wavelength features as non-linear absorption is not limited by optical diffraction. However, they are too expensive, 3–6 times as nanosecond CO_2_, excimer, and Nd:YAG systems.

### Selective laser melting and sintering (SLS)

This technique uses the powders with a high purity and properties similar to those obtained by traditional fabrication in the sintering so that it is advantageous over other 3D printing techniques. SLS is also used to write metal patterns onto polymers (e.g., PDMS), which has great potential in the design of biosensors. A variety of materials including metals, ceramics, and polymers, which are typically proprietary with poorly characterized surface properties, are used. Finer particles are used to produce accurate and smooth parts, but difficult to spread and handle. By contrast, larger particles facilitate powder delivery and process but hinder surface finish, resolution, and layer thickness. However, the obstacle of fabricating fluidic devices by SLS is that it is very difficult to remove the powder precursor from small cavities.

### Fused deposition modeling (FDM)

FDM can print a large number of cheap and biocompatible polymers, such as acrylonitrile butadiene styrene (ABS), poly-lactic acid (PLA), polycarbonate, polyethylene terephthalate (PET), polyamide, and polystyrene owing to its advantages of safety, reliability, easiness in the use, office friendness, low price, low levels of fumes from polymer at high temperatures, and no requirement of post-processing. FDM of liquid precursors, such as metallic solutions, hydrogels, and cell-laden solutions, has been implemented in the manufacture of LEDs, batteries, strain gauges on flexible substrates, antennas, interconnects, and electrodes in biological tissue. However, the structural strength of FDM printed structures is low and prone to compressive stress fracture because the extruded material immediately hardens and the adjacent layers are not well fused. There is a trade-off between printing resolution and surface finish, and the smallest fluidic channel achievable (~100 μm) is still larger than those made by SL.

### Polyjet or multi-jet modeling (MJM)

**Fig. 1:**
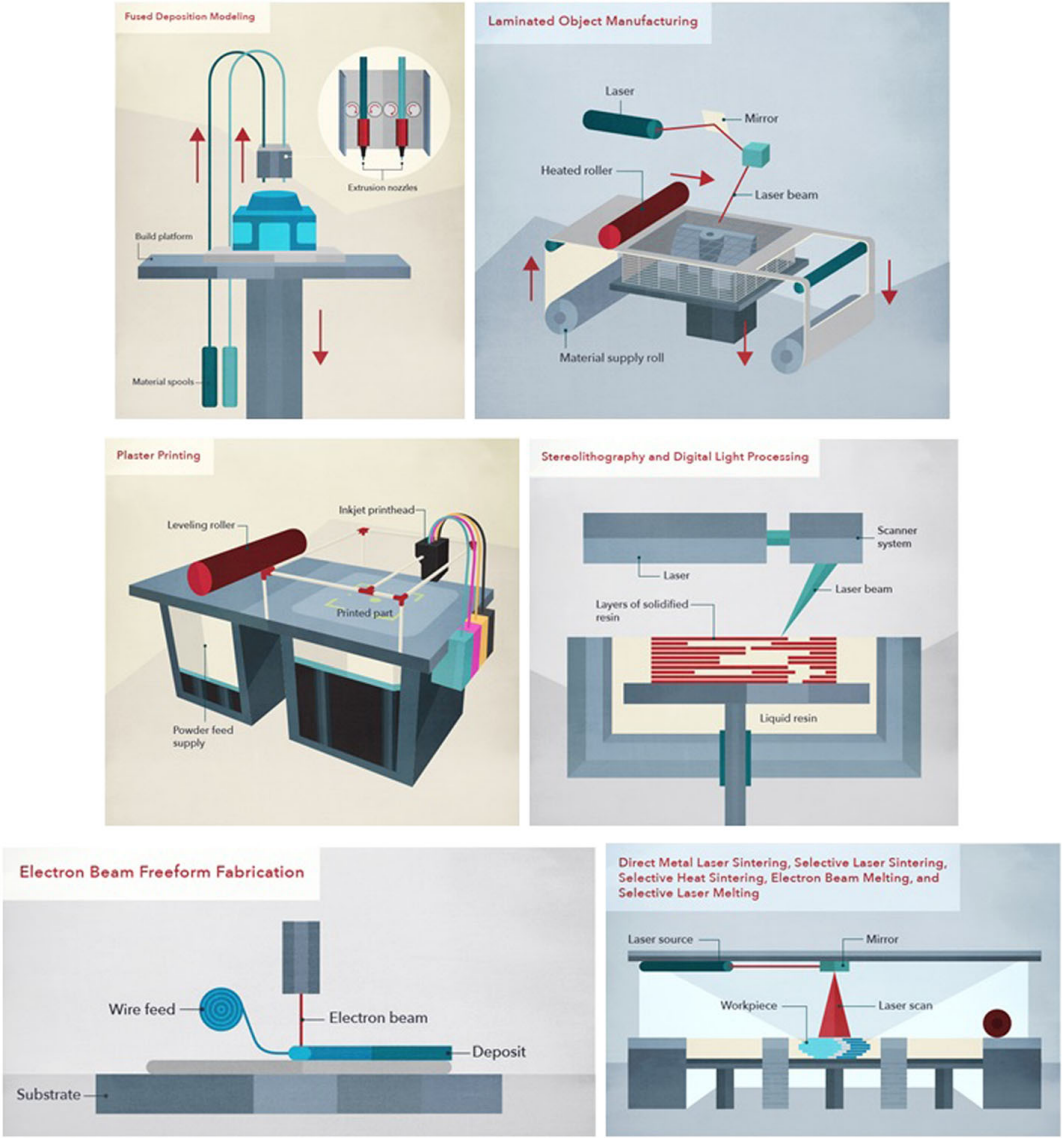
Diagram of various 3D printing techniques including fusion deposition modelling, laminated object manufacturing, plaster printing, sterolithography, electrom beam freeform fabrication, and selective laser sintering, with courtesy of CustomMade.com

MJM is attractive for fluidic applications because of high resolution and capability of printing multi-materials (over 100 different raw materials, including 22 from Stratasys, 38 from 3D System, and many ones used in the lab). Inkjet operates either in continuous or drop-on-demand (DoD) mode. The polyjet printer produces smooth features with the surface roughness of 0.47 μm in comparison to that of 42.97 μm in FDM. The inkjets can deliver simultaneously multiple materials with a wide range of properties (e.g., hard and soft plastics, elastomers) in different colors. However, the currently available materials are proprietary and expensive, and rigorous biocompatibility and bio-functionality investigations are required. The smallest printed fluidic channels are approximately 200 μm [42]. Comparison of these 3D printing technologies is listed in Table 1, and their diagrams are shown in Fig. 1 [24, 25]. Surface roughness induced van der Waals, electrostatic, and steric forces are unique to microfluidic flow. The induced shear stress may cause transient pores on cell membrane for complete cellular death and enhanced apoptosis [42]. In addition, silicone or mineral oil has been used to match the refractive index of fabricated devices in order to reduce the spherical aberrations for optical imaging.

**Table 1:** Comparison of fluidic channels manufactured by different 3D printing technologies

3D printing	materials	advantages	limitations	surface roughness	resolution	chip complexity
stereolithography	photosensitive resin/polymers	high resolution, good surface finish, little topological restriction	small volume (1PP), slow build time (2PP), high optical absorption required, cytotoxic, low throughput	~2 μm	25–300 μm < 1 μm for 2PP	high
selective laser sintering	powders of metal, polymer, ceramics	high resolution, fully automated	non-transparent, remaining powder precursor in small cavities	dependent on the powder size (on the order of 10–100 μm)	1–150 μm	moderate
fused deposition modeling	thermoplastics	cheap, ease of support removal, little topological restriction, fully automated	slow build time, low accuracy, non-transparent, poor gas permeability,	3–43 μm	100–400 μm	low
inkjet	photocurable polymers	fast build time, multiple materials	tedious removal of support, low accuracy	< 1 μm	20–100 μm	high
bioprinting	bioink, hydrogels	multiple materials	low build time, low viscous solution, low accuracy	10–330 μm	5–100 μm	high

### Scaffold-removal method

**Fig. 2:**
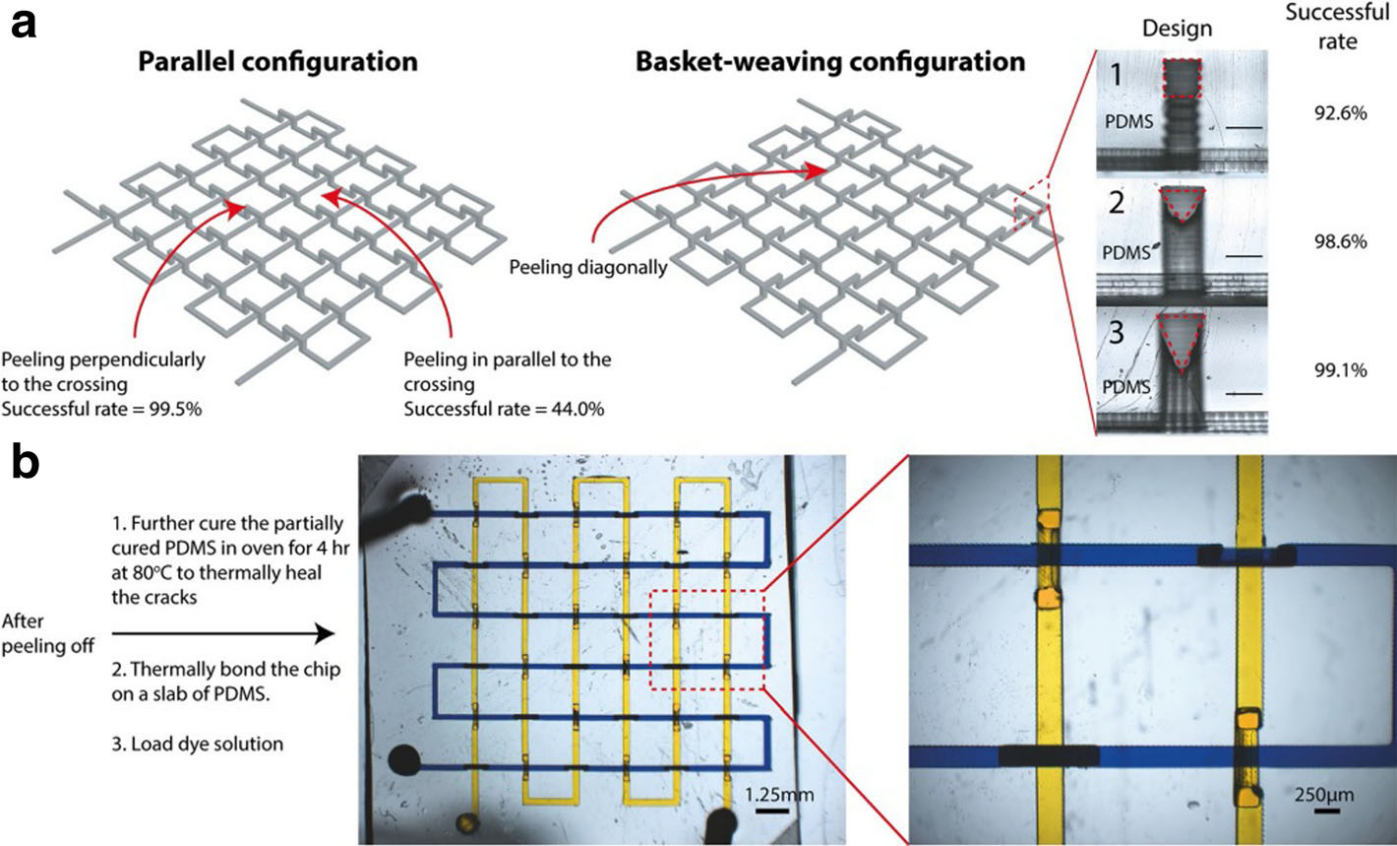
**a** Effect of the peeling direction and crossover features on the successful rate, scale bar of 250 μm, **b** image of the permanently healed 3D chip (basket-weaving configuration) loaded with yellow and blue dyes and magnified image of the dash line depicted region, with courtesy of [124]

Realistic 3D channels may not always be formed by one-step molding because the molding material and the master will be interlocked with each other. Peeling off the PDMS from the master under its partially cured state will generate a crack which will be self-closed afterward due to its elasticity and self-adhesion by further thermal curing. Oxygen plasma treatment followed by silanization is to coat a monolayer of fluorinated molecules on the 3D printed master to prevent PDMS from sticking to it. Heating at 130 °C is able to remove the unreacted additives and monomers inside the printed master. Such “heating–plasma–silanization” strategy allows researchers to fabricate 3D fluidic chips easily without bonding and alignment repeatedly and the clean room. Print-and-peel (PAP) techniques, printing the masters directly for casting polymers and adding 3D components onto the masters for single-molding in the bulky slabs, are facile and expedient in prototyping fluidic devices with regular office equipment (see Fig. 2). Ink or toner is deposited on the surface of the smooth and non-absorptive substrate (e.g., overhead transparency films) leaving positive-relief printout features by LaserJet or solid ink printer. PAP has been utilized to fabricate polymer micromixers, capillary electrophoresis, valves, gradient generators, optical waveguides, microelectrodes for μTAS. While the channels fabricated by photolithography have almost rectangular cross sections, those made from LaserJet- and solid-ink-printed masters have trapezoidal and round-bottom cross sections, respectively. The smallest lateral feature size reproduced on printed masters is around 100 μm while the heights of the features do not exceed 15 μm. However, the durability of the printed masters needs further improvement for mass production.

Although the fluidic devices printed by inkjet-printed are inexpensive, they are limited to planar channels on the glass surface. Alternatively, double helix channel is also possible. Strands in such shape are first manufactured and anchored, and their inner surfaces are separated by 2 mm through the posts on both ends. The PDMS prepolymer is then cast around the double helix structure, and the mold is then manually extracted following the curing process to create helical channels (500–1000 μm). The mold material inhibits full curing of the PDMS at the mold/PDMS interface [43]. However, such manual extraction method is only feasible for certainly shaped scaffolds.

Most subtractive methods can only remove material from the surfaces and are inappropriate for fabricating complex fluidic chips. Materials such as carbohydrates, hydrogel, metals or polymers are used as sacrificial templates and removed from the solidified polymer. The scaffold plastic polymer (e.g., ABS) is suspended into liquid PDMS and dissolved using a PDMS-inert solvent (e.g., acetone for 12 h) after curing the PDMS, leaving an empty cavity inside the PDMS. The swelling ratio of acetone for PDMS is as low as 1.06 [15]. Scaffold-removal method is powerful and versatile in creating multilevel and intricate fluidic channels. Integrating external elements directly in the fluidic device is desirable for LOC, but difficult to achieve using standard PDMS fabrication methods. Heating coils, RF circuitry or electronic components are also able to be embedded. However, reliably clearing a sacrificial material from an enclosed channel is limited by diffusion and quite challenging to producing arbitrary microfluidic networks in a single step. In addition, harsh condition, such as high temperatures for creating or removing [44] and applying heavy swelling for pulling out the template [45], are the limitation of this approach.

**Fig. 3:**
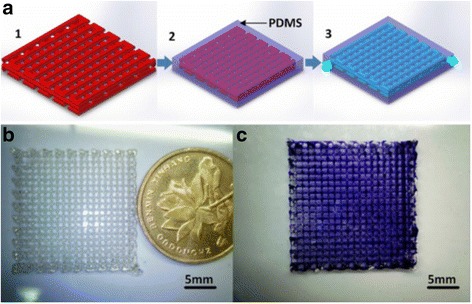
**a** Fabrication procedures, 1: direct 2D or 3D sugar structures printing; 2: pouring PDMS on the structures; 3: removing sugar structures to obtain fluidic chips without further sealing, **b** printed 3D sugar structure, and **c** a 12-layer 3D microvascular network, with courtesy of [125]

Fused sugar has an advantage as a sacrificial template to fabricate smooth channels owing to its efficiency and dissolution [46, 47]. Maltitol is selected due to it stable melt status, suitable surface tension, and high water solubility. The process is usually within 5 min. The diameter of printed sugar filaments is affected by nozzle diameter, air pressure, printing temperature and speed [48]. PDMS is then cast onto the sugar structures. Such process is repeated for each layer. Finally, PDMS is solidified at 85 °C for 25 min. Fluidic chips are immersed into hot water to dissolve the sugar lines without further sealing (see Fig. 3). Low cost is a significant advantage of sugar printer (~ $800). However, the structure without appropriate supporting materials may restrict the design complexity because of the structure collapse occurred between the large junction space of different channels due to surface tension and gravity of hot sugar filaments. Similarly, PDMS fluidic devices can also be fabricated with 3D wax jetting by a glass nozzle and a lead zirconate titanate (PZT) actuator [49].

**Fig. 4:**
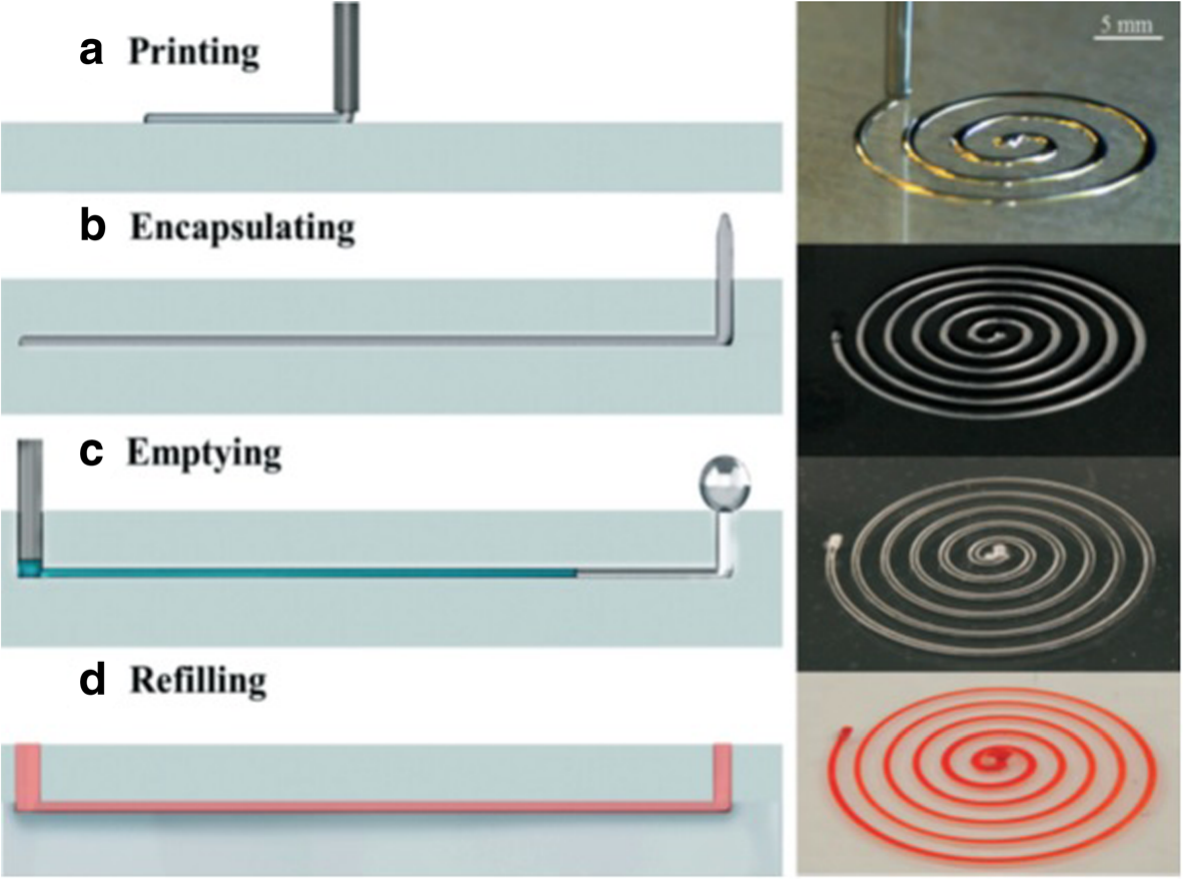
Schematic diagrams and images of fabricating 3D microchannel using liquid metal: **a** printing the liquid metal, **b** encapsulating the printed pattern in a polymer, **c** withdrawing the metal, and (**d**) refilling with red dye solution, with courtesy of [126]

One of the liquid metals in the use is EGaIn, a eutectic alloy of gallium (Ga) and indium (In) in a 3:1 ratio by weight with a melting point of 15.5 °C. Because of the passivating oxide skin thin shell forms instantaneously on the metal surface at room temperature. 3D printing is done using a micropositioning stage and a pneumatic air dispensing from a syringe. The printed structures are small due to the short distance between the nozzle tip and the substrate (<100 μm), and the movement of the nozzle generates stresses that could “neck” the metal filament spanning across this stand-off distance. Casting and curing polymer onto the printed features define the microchannel wall. After the printing, the EGaIn can be withdrawn using electrochemistry (e.g., 1 M HCl) because the drop and a bead of liquid metal at the other end act as anode and cathode, respectively, which is less harsh than acid. The metal bead also lowers the Laplace pressure at the outlet, making it easier for withdrawing the liquid metal (see Fig. 4). The height of structures printed is limited by the stability of the oxide skin (e.g., ~4 mm for EGaIn) especially in the embedding due to shear forces.

**Fig. 5:**
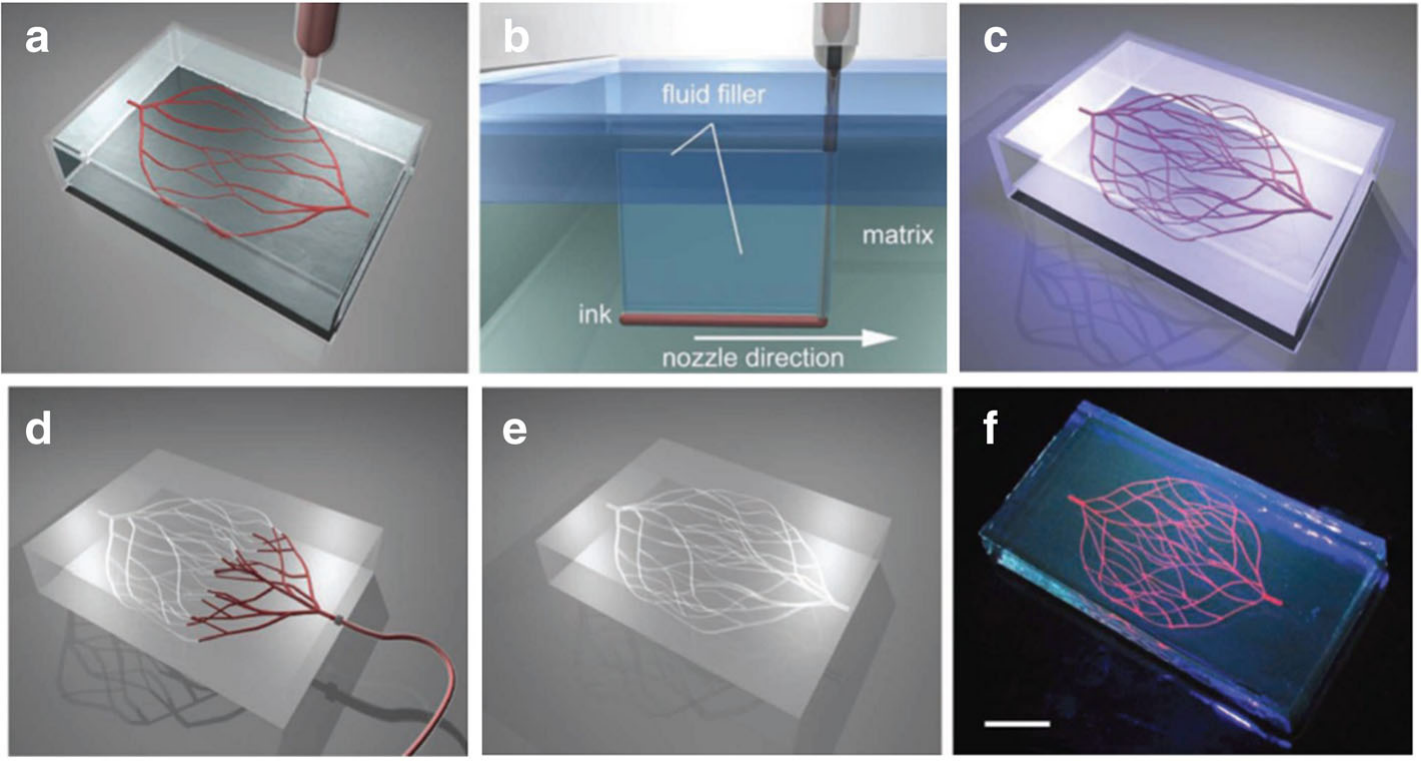
Schematic diagram of omnidirectional printing of 3D microvascular networks in a hydrogel reservoir: **a** deposition of a fugitive ink into a gel reservoir to pattern hierarchical, branching networks, **b** filling the voids induced by nozzle translation with liquid that migrates from the fluid capping layer, **c** yield of a chemically cross-linked, hydrogel matrix by photo-polymerizing the reservoir, **d**, **e** exposure of the microvascular channels by removing the liquefied ink under a modest vacuum, and **f** fluorescent image of a 3D microvascular network (scale bar = 10 mm), with courtesy of [53]

Direct ink writing (DIW) is an attractive method for creating 3D microvascular structures [50–52]. A fugitive organic ink is patterned into the desired motif, encapsulated in a thermally or photocurable resin, and subsequently removed by liquefaction to yield uniform microchannels interconnected. Omnidirectional printing (ODP) is a new variant of DIW and obviates the need for layer patterning (see Fig. 5) [53]. The deposition nozzle is inserted into a photocurable gel reservoir, which is formed by pouring 25 *w*/w% Pluronic F127-diacrylate into a silicone mold at 4°C and slowly solidifying at room temperature and can physically support the patterned features. Air pressure extrusion is applied to print the fugitive ink filaments, whose size is linearly proportional to applied pressures. As the deposition nozzle translates through the reservoir during printing, void space is generated locally and immediately filled by a 20 *w*/w% Pluronic F127-diacrylate fluid on the top of the gel reservoir because the liquid filler has identical chemical functionality, but a significantly lower viscosity than the photo-polymerizable reservoir. Afterward, the gel reservoir and fluid filler are solidified via photo-polymerization under 365 nm UV light for 5 min to form a mechanically robust, chemically crosslinked matrix. Because the fugitive ink filled in the printing nozzles from 10 μm to 200 μm in diameter (an aqueous solution of Pluronic F127 in 23 *w*/w%) has not been chemically modified and pronounced shear thinning behavior (shear modulus >10 kPa), it can be removed by liquefaction below the critical temperature (< 10°C) under a light vacuum to yield the microchannel network. This approach allows omnidirectional freeform fabrication of 3D biomimetic microvascular networks composed of a hierarchical, 3-generation branching topology with various diameters from 200 to 600 μm [53].

### Bioprinting

A large number of biomaterials (e.g., living cells and growth factors) can be directly printed using a 3D bio-printer. Fabrication of complex and heterogeneous structures using multi-head systems is relatively slow, which limits their use for cell-laden construct. Using integrated bioprinting-fluidics technology, the flow of different bioinks or even ECM components can be integrated into fibers or droplets, which opens new routes for creating realistic tissue fibers on demand. Robust hydrogels can be extruded through the dispenser, but soft gels are in the form of continuous polymer strands ideal for building constructs. 3D cell encapsulation is advantageous over conventional 2D cell culture in the cellular morphology, proliferation, and gene and protein expression because of improved cell–cell contacts and cell–matrix interactions [54]. Inkjet can print bioinks consisting of cells, DNA, and biomaterials [55, 56], while FDM is able to create 3D multi-material scaffolds for cell seeding [57]. Mechanical forces during the bio-printing are determined by extrusion speed, nozzle diameter, the viscosity of hydrogels, and temperature. The shear forces applied on embedded cells increase with the decrease of nozzle diameter and the increase of the extrusion speed and the viscosity of hydrogels by decreasing the chamber or nozzle temperature [58]. Simultaneous extrusion of an alginate and a calcium ion solution through the inner and outer needles, respectively, of the coaxial extruder permits the formation of a gel fiber at the tip and lays it according to the design. The optimal formulation is 4% *w*/*v* alginate and a 0.3 M solution of CaCl_2_. Such method obtains macroscopic and porous 3D structures with single fiber thickness in the order of 100 μm. However, high concentrations of crosslink materials (>2%) have a negative impact on the cell’s salt balance. The use of two independently cross-linkable hydrogels allows the tuning of mechanical properties of the cell-laden fibers to mimic the morphological and mechanical features of native tissue. The concentration and densities of two different hydrogel-precursor polymers are adjusted for a higher printing resolution of cell-laden fibers and the ideal microenvironment for cell spreading and organization. Overall, the cells should be robust for physical forces (e.g., shear stress and pressure) and biological stressors (e.g., the presence of toxins, enzymes, and nonphysiological pH) in the bioprinting. Current 3D bioprinting approaches involve biomimicry, autonomous self-assembly, and mini-tissue building blocks [35].

### Paper-based fluidic device

Paper-based fluidic devices are recently developed and have significance in the simple fabrication for mass production, low cost, ease of transportation, storage, and disposition, simple liquid motion without excessive equipment, but the high efficiency. Fabricating a micro paper-based analytical device (μPAD) by wax printing involves only two main procedures: printing wax patterns on the paper and then melting the wax across the paper to form hydrophobic barriers both laterally and vertically to prevent the fluid mixing passing through the device. The low production cost and complexity levels in the manufacture of μPAD are appropriate for prototyping at a large scale. Among the numerous techniques utilized to create channels on hydrophilic paper, solid ink (wax) printers are the most promising one for smooth features [59] while the granular structure of the LaserJet toners is conspicuous on the replicas. The relatively low melting point of the wax prevents the cast PDMS from curing at elevated temperature. The lateral dimensions achievable with office-grade solid-ink printers are about 200–300 μm while that of office-grade LaserJet is 50 μm [60]. Although the features are not accurate and sharp at the edges, they are sufficient in detecting substances due to color change in the test assay. Overall, μPAD may develop to rapid, cheap, flexible, and reliable devices for clinical emergency and large-scale use [59]. This fabrication method is quite new, and the control over flow rates, mixing, and interaction times between sample and reagents needs to be improved.

## Applications

Microfluidic systems are valuable tools in flow cytometry, cellular assays (e.g., cytotoxicity or cellular stress assays), cell sorting, manipulation, and imaging, molecular analysis, cell response to chemical and physical stimuli and tissue engineering because of precise control of small volumes of fluids over short distances. Array design provides the possibility of parallel measurement of a large number of samples. Some of the emerging applications of fluidic channels in biology and biomedical engineering are listed below to illustrate their technical advantages. These applications are summarized and listed in Table 2.

**Table 2:** Comparison of applications of 3D printed microfluidic channels

application	pros	cons	references
molecule & protein detection	various electrode materials integrated into microchannel; various measurement and functionalities; easy operation in all environments	fabrication complexity with increased number of embedded sensors, large fabrication error width in paper channel	[40, 59, 61, 128, 129]
cell deposition & simulation	at the resolution of individual cells, the possible molecular interactions between cells, 3D concentration of gradients, precise control of fluids, reduced reagent/sample consumption, robust and automated procedure	rather large fluidic channels, discrepancy between the printed and designed channel height	[19, 63–67]
bacterial communities	multiple population, no external force required, little physical damage to cells	hard to predict and control the flow behavior in a channel with varying curvatures, small bacterial concentration in the detection	[68–70, 130]
3D tissue constructs	precise control over various cellular microenvironment, easy formation of desired structures, high throughput, reproducible, multi-layer structures	trade-off between cell density of bioink and nozzle size	[51, 74, 78–82, 85–88, 90, 91]
organ-on-a-chip	accurate position of various tissue samples	throughput is limited by large components with intricate geometries	[15, 17, 92, 118, 131]
organ conformal biopsy	rich diagnostic information, continuous monitoring, direct coupling	unknown long-term effects for human	[94]
Milli- & micro-fluidic reactionware	rapid production and design optimization, quick and versatile material synthesis, high temporal stability	low output volume, inability at high pressure and temperature	[62, 95–97]

## Molecule and protein detection

A variety of electrode materials (e.g., carbon, platinum, gold, silver) can be easily integrated into microfluidic devices for various applications (e.g., neurotransmitter detection, NO measurement, oxygen tension in a stream of red blood cells) along with other functionalities (e.g., fluidic interconnects and membrane inserts) for molecule analysis (e.g., ATP via chemiluminescence). Essentially, the electrode fitting is removable and reusable. 3D printed fluidic channels not only change the strategy of research collaboration but also the perceived limitations of the biological experiment, where the spatial control of samples or cells is critical [61]. Biosensors could also be integrated and placed consecutively in the fluidic chips. Paper-based fluidic devices are biodegradable, cost effective in the disease diagnosis, and easy in almost all environments. The amounts of glucose and protein in the paper fluidic channels are proportional to the color change of each assay [59]. The smallest width of the printed hydrophobic barrier is 400 μm, and expanded to 1000 μm after melting.

### Cell deposition and simulation

**Fig. 6:**
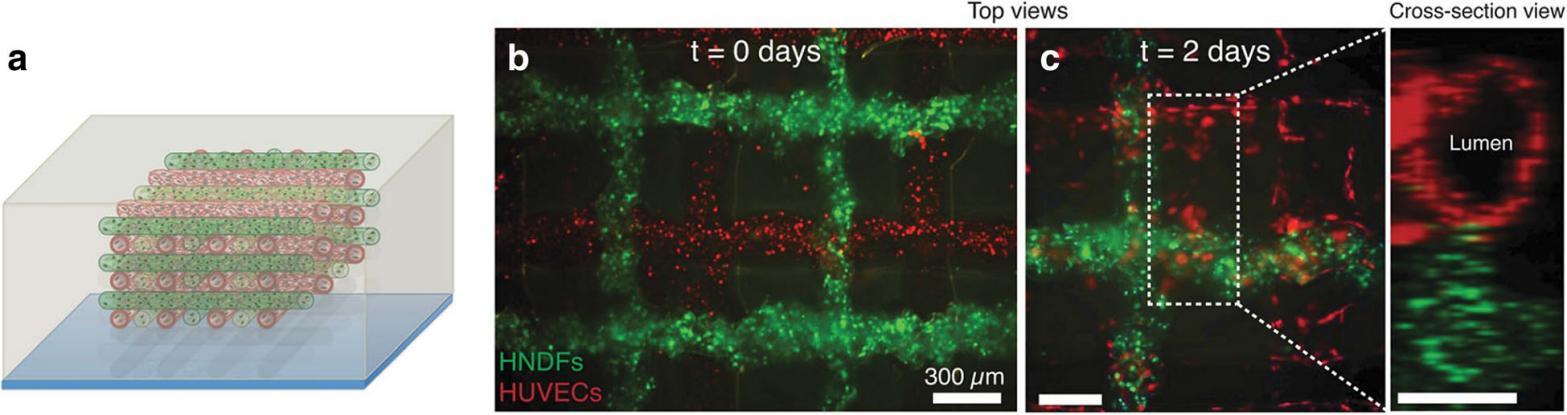
**a** Schematic view of red and green filaments with RFP HUVECs and GFP HNDF-laden GelMA ink, respectively, and (**b, c**) fluorescence images of an engineered tissue construct cultured for 0 and 2 days, respectively, with courtesy of [121]

The complicated topology of the 3D microfluidic network in the stamp makes it versatile to pattern multiple proteins and cells. However, the cells have limited migration and growth and stop dividing once they form a confluent layer between the stamp and the substrate [62], and continue to spread and divide once the stamp is removed. There are two levels of membrane structures: one as the channel plane open for contact with the substrate, and the other as vias connecting channels in the membrane to those in the slab. Autoclaving is necessary to improve the viability of cells inside the PDMS stamp [63]. Such deposition of multiple cell types and proteins in complex, discontinuous, well-defined patterns has great value because in vivo ECVs (tumor cells) attract and direct the growth of BCEs (capillary cells) for tumor angiogenesis and nutrients and oxygen supply. It makes 3D micromolding in capillaries (MIMIC) a powerful technique in investigating the differential and competitive attraction of capillary endothelial cells to different tumor cells, which can be developed to a simple, standard, and quantitative in vitro assay for evaluating the angiogenic potential (see Fig. 6). It can also be used in investigating the functional significance of tissue architecture at the resolution of individual cells, and the molecular interactions between cells that underlie processes of embryonic morphogenesis and formation of the blood–brain barrier.

Concentration gradients of soluble factors (e.g., growth factors, chemokines, and gas molecules) are essential for physiological and pathological processes in vivo. Thus, the generation of 3D concentration gradients has strong implications for tissue engineering and drug screening. There is a gradient of physical properties from central to the peripheral vascular tree. For example, the arteries closer to the heart are thicker and more compliant whereas arteries further along the vascular tree are considerably thinner and stiffer. Microfluidic technologies provide benefits over conventional cell culture and experimental systems because of the precise control of fluids (on the scale of fl and nl), cost effectiveness, reduced reagent/sample consumption, and robustness via automated experimental procedures [19, 64–66]. The mold fabricated using the 3D printing from a single material shows superior mechanical stability in comparison to photoresists on silicon. However, current fluidic channels are still quite large. The discrepancy between the printed and designed one is 30–70 μm at the channel height of 250 μm and increases significantly at the channel height of 100 μm [67]. Those below 50 μm are not applicable now.

### Bacterial communities

Microscopic printing also enables to organize multiple populations of bacteria within 3D geometry (e.g., adjacent, nested, and free-floating colonies). To investigate the behavior of small microbial aggregates (e.g., whole bacteria, cells, ATP, oxygen, and other essential biomolecules), a number of microfabrication technologies have been developed to confine bacteria within the fluidic devices, cavities, and liquid droplets for assaying antibiotic resistance and enzymatic activity. Such process of encapsulating cells often restricts mass transport, which is incompatible with growth and signaling between physically isolated populations. The resistance of one pathogenic species to an antibiotic can enhance the resistance of a second species by virtue of their 3D relationship. Moreover, this fabrication approach using bio-printing can define bacterial micro-colonies in animal hosts for infections development in vivo [68]. 3D printed chip by FDM is also suitable for bacterial cultivation, DNA isolation, PCR, and detection of an amplified gene using gold nanoparticle (AuNP) probes for early diagnosis with compactness and low cost.

**Fig. 7:**
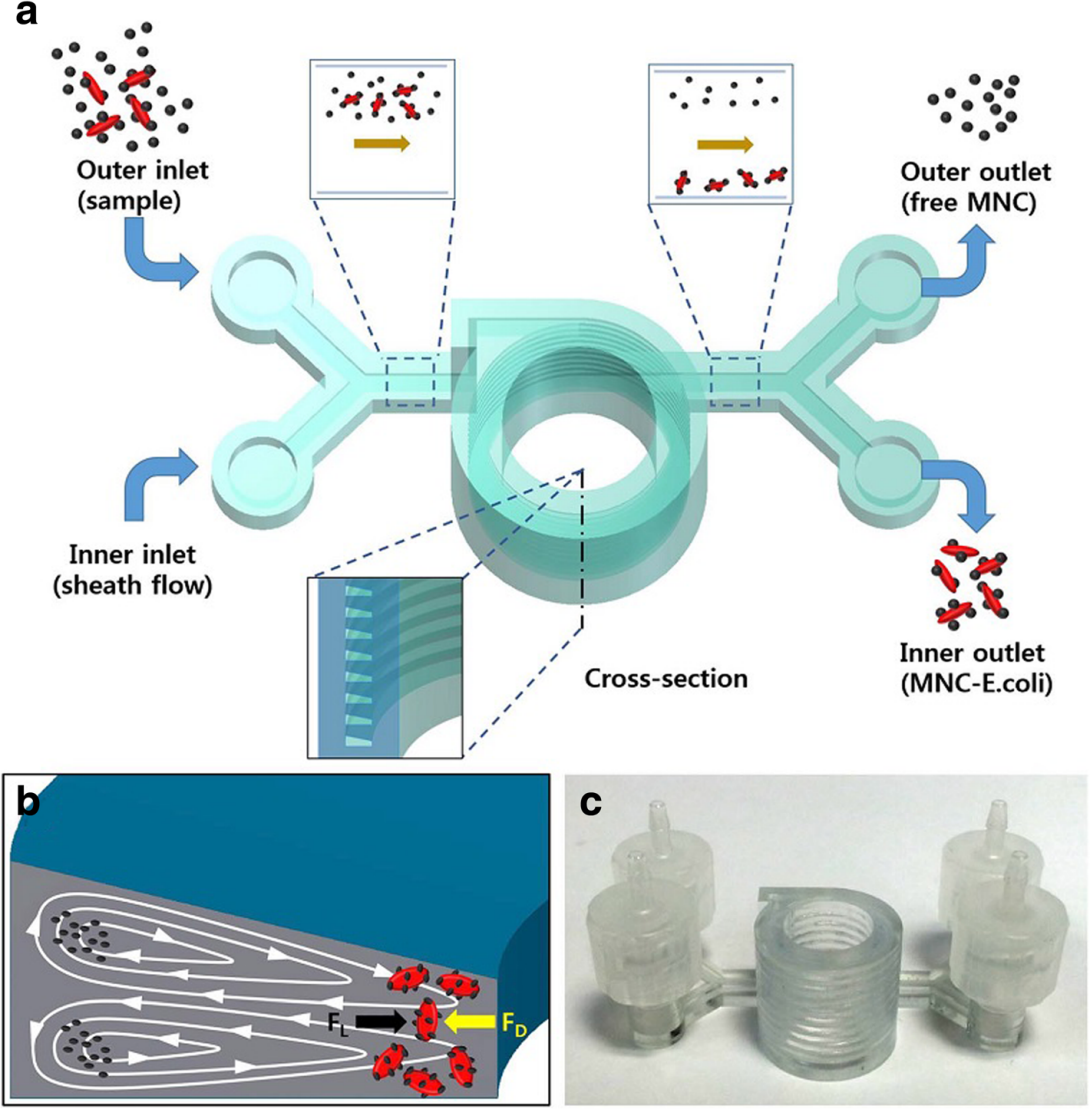
**a** Illustration of separating bacteria by inertial focusing, **b** Dean vortices in a channel with trapezoid cross-section, and **c** photograph of the 3D printed device, with courtesy of [70]

Because of fast proliferation, sensitive measurement of bacteria at the early stage is critical for preventing food-borne diseases [69]. Microbial cultivation-based detection is accurate and reliable as a golden standard method. However, its application is limited to laboratory measurements owing to the intensive consumption of time and labor. Size-based separation techniques are preferred because of no requirement of a complicated labeling procedure. The inertial focusing by Dean drag force has been successfully used to separate cells and particles in a 2D PDMS substrate with easy control of the operation condition, but no external force required and little physical damage to the cells. However, varying the curvature radius in a spiral channel with different Dean numbers makes the flow behavior difficult to predict and control. A helical microchannel around a cylindrical chamber was fabricated using stereolithography in a compact size with a constant radius of curvature (see Fig. 7). Large antibody-functionalized magnetic nanoparticle (Fe_3_O_4_) clusters are focused near the inner wall of the microchannel where Dean drag force and the magnetic lift force proportional to the particle volume are balanced. To improve the separation, a sheath flow is introduced to push the particles to the outer wall of the microchannel and help their trapping in the strong Dean vortex cores. The detection limit is 10 cfu/mL for *E. coli* bacteria in buffer samples and 100 cfu/mL in milk due to the less capture efficiency by the presence of interferents [70].

### 3D tissue constructs

Biological tissues and organs have a large number of microvascular networks facilitating oxygen and nutrient delivery and waste removal from the surrounding cells. Mimicking such network is of considerable importance for self-healing [50, 71], replacing a damaged native blood vessel, tissue engineering [72], organ printing [73], body-on-a-chip [74], tubulogenesis and vascular morphogenesis [51, 75], cardiovascular pathology, pharmacological modeling, drug testing (e.g., functionalization of biomaterials with proangiogenic agents), and biomedical devices. For example, skin mimicking samples with healing agents in the synthetic microvascular networks could repair the damage repeatedly [50, 76]. Numerous techniques have emerged to induce the formation of vascular structure within tissues and can be classified into either pre-vascularization-based or vasculogenesis- and angiogenesis-based types. Due to a lack of effective vascularization, there are severe limitations in the clinical development of vascularized complex 3D tissues, particularly those large vital organs (e.g., liver, kidney, and heart) [77]. While the pre-vascularization techniques provide readily available channels for immediate perfusion of growth media or blood and fabrication of larger blood vessels, they are not suitable for vascular capillary beds with cascading bifurcations down to a few micron sizes. The vasculogenesis- and angiogenesis-based approaches, on the other hand, provide very limited control over the temporal and spatial factors, require days to weeks before cells can organize and grow perfusable lumens, and are not suitable for formation of vascular structures for suturing and anastomosis with the host vasculatures. The combination of hydrogels, microfabrication techniques, and microfluidic systems may overcome the challenges of developing an artificial microvasculature [78–80] by offering precise control over various cellular microenvironment including fluid flow, chemical gradients, localized ECM as well as the microenvironmental cues such as mechanical properties (e.g., stiffness), chemical properties (e.g., ligand density and orientation), and topographic features (e.g., different cell substrate affinity). The vascular channel is constantly perfused and resides on a biologically relevant and porous matrix where other cells can be introduced easily to form the desired structures. The embedded live cells and growth factors along with biomaterial channels at precisely controlled locations to mimic the native tissue architecture can facilitate the delivery of nutrient-laden fluids and promote cell viability [81, 82]. It has a great potential in tissue engineering because various functional tissues can be fabricated with appropriate structures and cell compositions (e.g., EC, SMC, fibroblasts, progenitor cells or various stem cells) in various sizes, high throughput, and reproducible fashion [83]. However, the fabrication of microvascular networks composed of complex, hierarchical 3D architectures is still quite challenging. Building appropriate vascular structure is critical to vitalize thick tissue which has difficulties in survival and proliferation due to diffusion limitation over a few hundred micrometers [84].

**Fig. 8:**
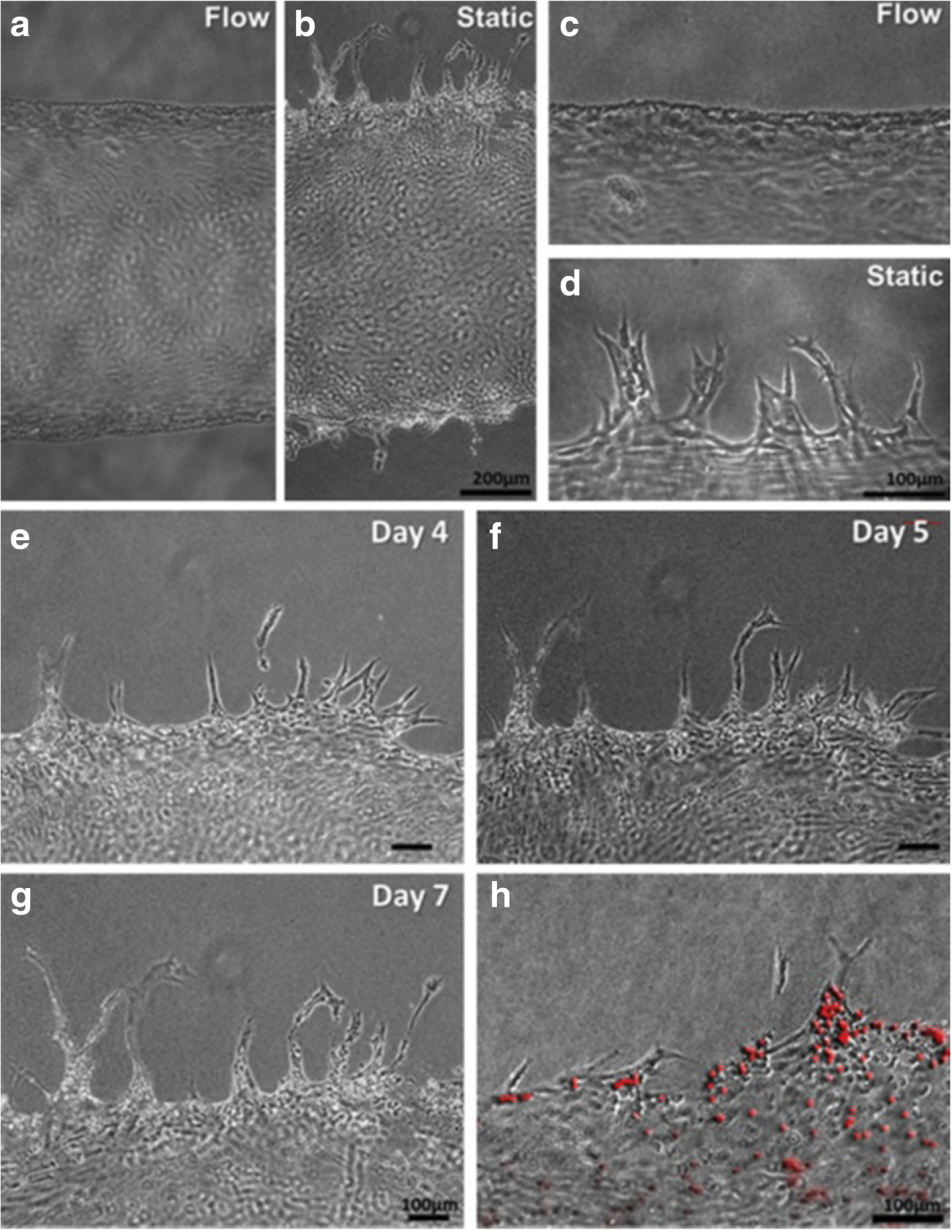
Morphology of HUVECs on the vascular channel edge in **a**, **c** dynamic culture and **b**, **d** static culture on Day 5, **e**-**g** the sprouts budded from the channel wall extending during culture and maintaining filopodia-like protrusion on the tip in the static condition, and **h** Luminal structure of sprouts as confirmed by the injection of fluorescence microbeads (10 mm), with courtesy of [85]

Culturing under dynamic conditions could sustain cell viability deep inside the scaffold. During the incubation process, HUVECs capture within the gelatin sink down slowly and attach to the inner surface of the channel [85]. HUVECs cover 70–80% of the inner surface area of the fluidic channel on Day 0. The cells proliferate and cover the entire inner surface within 2–3 days. Under the flow culture condition, HUVECs on the channel wall are elongated and aligned along the flow direction over time. In comparison, HUVECs cultured in the static condition escape from the channel edge, actively invading into the collagen scaffold and forming angiogenic sprouts (see Fig. 8). The sprouting initiates on Days 3–4 all over on the channel wall and extends up to 400 μm on Day 7. As the sprouts continue to invade into the collagen matrix, they become longer, contain progressively more cells, and begin to branch. Stereotypical sprouting morphology is observed in these sprouts, presenting thin filopodia-like protrusions at the sprout tips. Frequently, cell migration into the collagen matrix occurs exclusively by angiogenic invasion and sprouting.

Among the most extensively investigated methods for the effective introduction of angiogenesis in vivo is the control and regulation of the spatial and temporal distribution of common angiogenic growth factors (GFs), such as concentration gradients of VEGF, in a cell-laden or cell-seeded hydrogel in a microfluidic device. The endothelial cells (ECs) tend to migrate from the region in low-GF concentration toward that in high GF concentration, thereby aligning themselves into well-organized structures and enhancing the capillary-like tubular structure formation [77]. Hollow, calcium-polymerized alginate tubes can be easily patterned using 3D printing techniques. Its diameter can be precisely controlled in the range of 500–2000 μm by changing the flow rates or nozzle speed. The structural rigidity of these constructs allows the fabrication of multi-layered structures and maintenance of their hollow form without causing the collapse in lower layers [86].

**Fig. 9:**
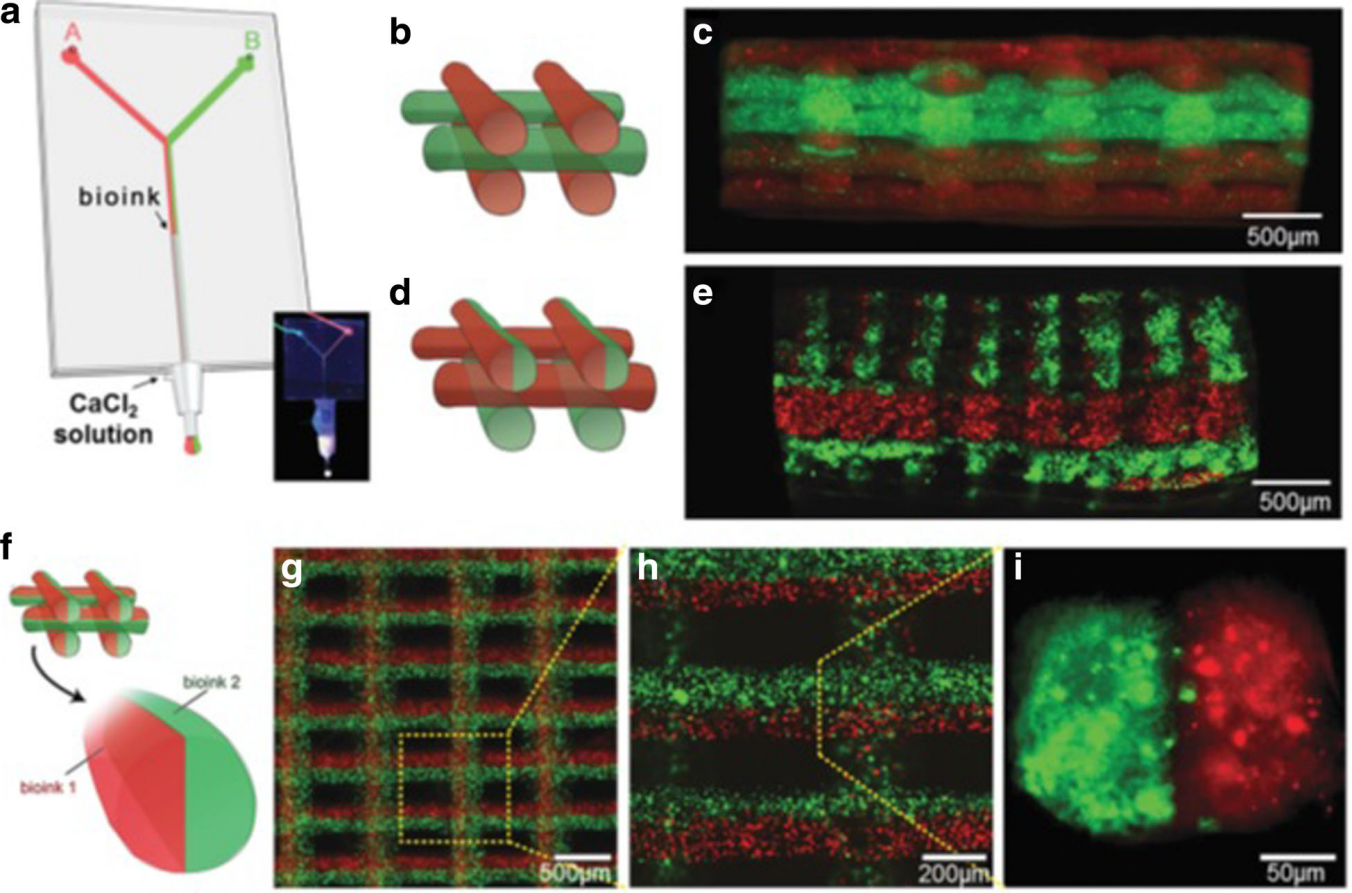
**a** A microfluidic system with the bioinks flow containing red and green fluorescent beads, photograph (inset) of the coaxial needle system with a “Y”-shaped microchannel, the illustration and fluorescence image of 3D construct with **b**,**c** alternate, **d**,**e** alternate/simultaneous, and **f**–**i** simultaneous deposition, with courtesy of [88]

The development of highly organized and functional 3D tissue constructs is still challenging. Precisely positioning different types of cells and biomaterials to resemble the in vivo environments is a major problem despite significant advances in 3D bio-printing [87]. Cell-responsive biomaterials are required to enable spreading and migration of the cells, printing low-viscosity bioinks for the use of a smaller nozzle and faster dispensing speed for a higher printing resolution and shorter fabrication time, and fast gelation process to support the cells located inside and outside of the printed scaffold and to create thick constructs with high cell viability. High-density cells (10^7^ cells/mL) within the polymeric solution can reduce the possible shear stress applied to the cells during the bio-printing. Furthermore, the fluidic platform incorporated to a 3D printing system can rapidly deposit multiple materials through an extrusion system and precisely switch between different bioinks and patterns, which allows the creation of heterogeneous 3D structures in the improved resolution and efficiency (see Fig. 9). For example, low concentrations of GelMA hydrogels (<5% *w*/*v*) with a low degree of acryloyl modification show spontaneous organization of encapsulated cells, such as human mesenchymal stem cells (hMSC) and ECs, in comparison to the high concentration (>10% w/v GelMA) with a high degree of acryloyl modification [88].

There are significant differences in cell behavior between 2D and 3D models in protein expression and gradients, drug response, cell migration, morphology, proliferation, and viability [89]. Most 2D cells float from the culture dish while 3D cell spheroids in the hydrogel are still maintained within the constructs. The metabolic activity in 3D and 2D Hela culture with the addition of paclitaxel is 0.47 and 0.06 times, respectively, in comparison to the control. It shows that 3D cell/hydrogel constructs are important in supporting the long-term proliferation of a large number of cells. Hela cells in the 3D model show higher MMP protein expression and chemo-resistance than those in the 2D culture [90]. The differences in gene expression patterns of 2D vs. 3D are likely due to the matrix composition and stiffness that ECs reside on (collagen vs. plastic surface). The heterogeneous distribution of biological-relevant proteins and growth factors in the tissue are essential for cell signaling, proliferation, and migration. Bioprinting can mimic the structure and function of in vivo cancer with 3D complexity (e.g., tumor heterogeneity, leaky and poorly organized vascular structure) in a biomimetric microenvironment in a high-throughput and reproducible manner at low cost. Stromal cells co-printed with tumor cells can naturally secrete ECM, growth factors, and hormones so that structural differences between the proteins used and the varying composition and materials in the exogenous scaffolds can be avoided. Histomorphological analysis showed adipose, stromal, epithelial, and carcinoma compartmentalization in the printed cancer models [91]. Living microarchitectures bioprinted from human cells are more realistic for creating cancer models without the cross-species difference which leads to the inaccurate prediction of the animal models in human testing [91].

### Organ-on-a-chip

**Fig. 10:**
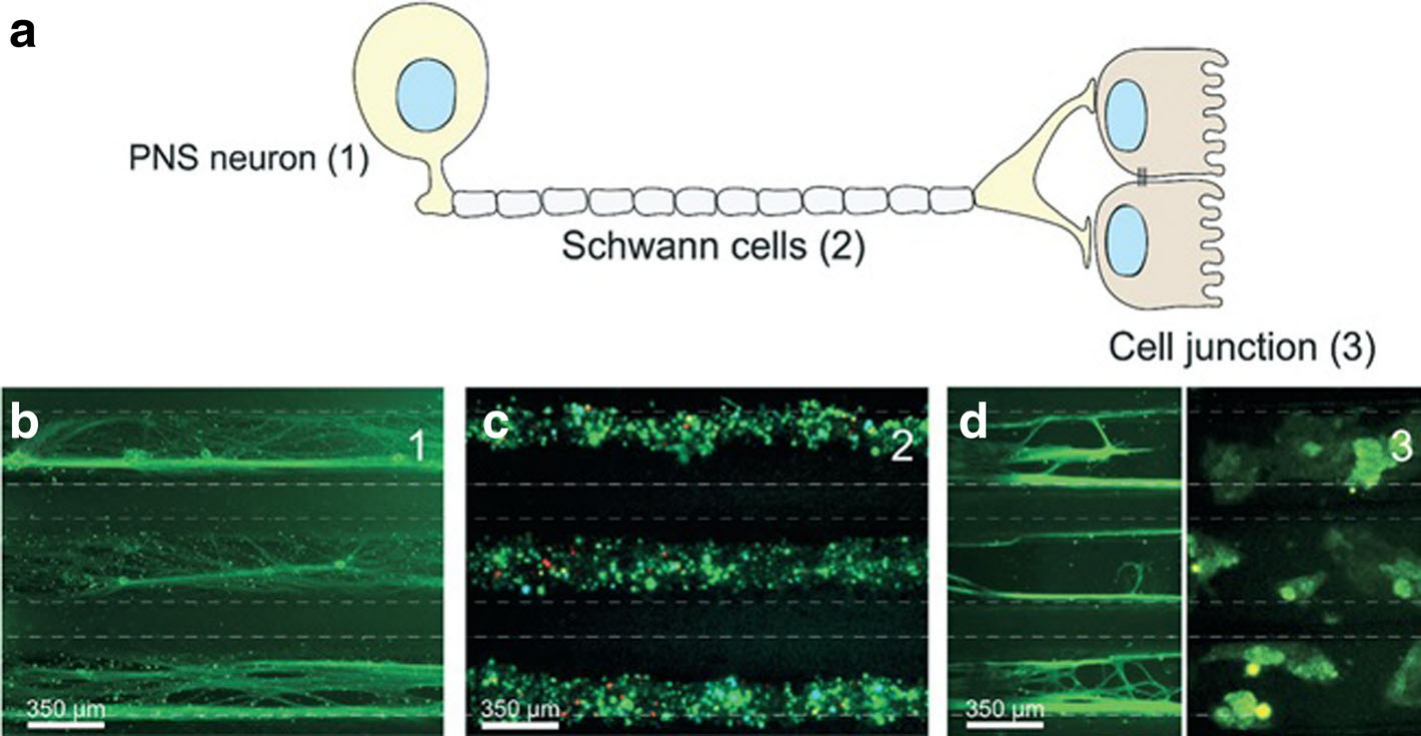
**a** Schematic of a peripheral 3D neural systems-on-a-chip consisting of peripheral nervous system (PNS) neuron, Schwann cells, and cell junction, micrograph showing three parallel microchannels of **b** PNS neurons stained by green tau, **c** peripheral nerve fibres stained using tri-colour pseudorabies virus **d** axon termini (green tau stained) and epithelial cells stained by gree tau and cytokeratin, respectively, with courtesy of [127]

Organs on a chip are microengineered tissues cultured in specifically designed and fabricated microfluidic bioreactors, supplying oxygen, nutrients, and growth factors and removing waste, to mimic the structure of human tissues better than current models. Because of the translational challenges associated with 2D monolayer cultures (e.g., inability of replicating higher order features and trajectories of human body) as well as ethical and economic concerns of small animal experiment, 3D printing of the next generation of microphysiological neural systems-on-a-chip (NSCs) can model human neurological diseases using stem cells in the construction of patient-specific NSCs, develop personalized neurology, and facilitate the preclinical drug screening with more flexibility, robustness, and efficiency in controlling and monitoring system parameters [15]. 3D printing affords repeatability and robustness in multi-layer and diverse-material (e.g., conductive materials as the substrate for stimulation and monitoring) fabrication for interweaving biology with scaffold and functional materials directly using the anatomical geometry obtained from the medical diagnosis. Micro-extrusion is more popular in developing NSCs because it is compatible with cell suspensions, cell-laden hydrogel, and thermoplastics. Microfluidic NSCs can model neurite outgrowth, fluid handling for perfusion, convective flow of nutrients and biochemical cues (e.g., cell migration, cell signaling, and gene expression), and mechanical actuation (e.g., shear stress and dynamic scaffold deformation). Laminar flow inside it allows the generation of complex and highly controllable fluid flow regimes. The bioreactor can maintain the viability of tissue constructs and accelerate tissue fusion, remodeling, and maturation. However, the throughput is limited by large components with intricate geometries. In compartmentalized NSCs, the co-culture of multiple cell types, media, and biochemical cues allows the investigation of cell-cell interaction in tissue self-assembly and circuit mapping (see Fig. 10). In comparison, hydrogel NSCs have more flexibility to design heterogeneous tissue by including multiple cell types, crosslinking hydrogels with different ECM compositions or different cell-laden hydrogels.

Another important platform is liver-on-a-chip for long-term culture (e.g., 30 days) of 3D human HepG2/C3A spheroids encapsulated with GelMA hydrogel for drug toxicity assessment in a bioreactor with continuous perfusion and in situ monitoring of the cellular functionality by analyzing the concentration of secreted biomarkers [92]. Culturing the cell spheroids could enhance homotypic cell-cell interactions with aggregated hepatocytes and improve functional outcomes. The toxic response in such hepatic construct was found similar to that of animal and in vitro models. Further investigation is required to yield cells with phenotype and functions similar to those of mature hepatocytes (e.g., drug metabolism, bile formation, and production of blood clotting factors and glucose). The other developed and successful models are for the blood-brain barrier, lung, intestinal [93].

### Organ conformal biopsy

Conformal microfluidic devices work as a minimally invasive “biopsy” for isolation and profiling of biomarkers from whole organs within a clinically relevant interval. 3D printing exhibits compatbility with conformal manufacturing of next-generation microfluidic devices and medical imaging technology for whole organ healthcare (e.g., organ assessment) because of a major advance in microfluidics and the direct coupling of the device to the surfaces of whole organs. The samples continuously isolated by the 3D printed organ-conforming microfluidic device provide rich diagnostic information, such as biomarkers of ischemic pathophysiology and metabolic activity. This achievement could shift the paradigm for whole organ preservation and assessment, thereby relieving the organ shortage crisis through increased availability and quality of donor organs [94].

### Milli- and micro-fluidic reactionware

The rapid realization of configurable and scalable reactors is highly desired in chemistry. The high surface area-to-volume ratio and precise control of the reaction environment are critical for LOC, but 3D printing technologies have been overlooked due to a perceived limitation of resolution. Reactionware is classified as nano- (1–100 nm), micro- (100 nm to 1 mm) or milli-fluidic (1–10 mm) devices according to the dimension of reactor [95]. Due to the ease of micro- and milli-reactionware fabrication, 3D printing facilitates rapid production turn-around, iteration and optimization of design based on experimental data at low cost once faults or errors using composite catalyst-silicone materials are found. Thus, it is straightforward to evolve the design of milli-fluidic channels in terms of geometry, inlets, outlets, and sizes, print in an appropriate material, and perform organic, inorganic or materials syntheses in one day, which allows versatility in the design and use of specific reactionware for experimental users [96]. Several chemical reactions, such as organic synthesis of an amine by two-step reductive amination and subsequent alkylation of the secondary amine, the inorganic synthesis of large polyoxometalate clusters, and the controlled synthesis of gold nanoparticles, have been efficiently carried out in 3D printed reactionware devices [62]. Nearly monodisperse silver nanoparticles have been synthesized employing miniaturized continuous flow oscillatory baffled reactors (mCOBR) employing additive manufacturing with higher temporal stability and superior control over particle size distribution than tubular flow reactors [97].

## Discussion

In order to push the commercialization of microfluidic channels, “killer apps” are necessary despite great potentials shown in various applications. Specific tasks, including the standard user interfaces, simple control on the microfluidic systems, and commercial manufacture, should be resolved. 3D printing has attracted attention in fabricating fluidic networks due to its automation, assembly-free, low costs, and continuously improved resolution and throughput. Microfluidic channels have great potentials for LOC (e.g., chaotic mixers, reagent and buffer reservoirs, fluid homogenizers). But they have limitations in terms of hardware, resolution, large channels size, resin versatility, overall device dimensions, the lack of control over resin formulation, subsequent surface and bulk chemistry, and prototyping system cost for uptake by “skill-less” biologists. With more commercial microchannel products, “killer apps”, maybe in the cell/protein detection and drug screening, will finally show up in a few years. Although 3D printing cannot substitute injection-molding at the mass production, it can produce small batches (from single to hundreds of parts) economically, efficiently, and environmentally (minimum waste and no tooling) for a smooth transition to injection-molding and easy design evolution, permitting a “fail fast and often” strategy in the device development based on the early and rapid empirical feedback [27]. 3D printing will make most PDMS and plastic molding in research laboratories but cannot completely replace the photolithography. Printed fluidic devices can dramatically reduce the barrier of sophisticated designs and positively disrupt the developmental cycles [98]. Although the current resolution of 3D–printers does not match that of soft lithography, 3D printing provides a new route of integrating user interfaces and embedded controls. The ability to clear the uncured or partially-cured resin (in SL) or sacrificial polymers (in MJM) from the 3D printed channels is important for the fabrication resolution. However, the development in desktop SL and MJM devices, photo-resins, multi-material 3D printing as well as the expiration of patents and the emergence of competing platforms are ushering in significantly improved resolution, throughput, and functionality [39]. Systems with <10 μm resolution for <$10,000 are not far-fetched; for example, a current system of $5000 can achieve resolution of 25 μm [99]. Models up to 43 mm×27 mm×180 mm at the speeds of 20 mm/h in the height were fabricated using a commercial 3D printer costing $2300 with 500 mL of the clear resin of $138 for any design complexity [100]. Oxygen inhibition of free radical polymerization in air results in incomplete cure and surface tackiness and is a widely encountered obstacle. Continuous liquid interface production (CLIP) is enabled by creating an oxygen-containing thin uncured liquid layer between an oxygen-permeable window and the cured part surface with the thickness of tens of μm by judiciously selecting the photon flux and resin optical and curing properties [101]. Subsequently, the resin polymerization speed could be increased to hundreds of mm/h. Plenty of work has been carried out in the open fluidic channels, which is harder than printing templates, but easier than printing enclosed channels by removing the uncrosslinked resin. The use of both additive and subtractive methods could lead to new devices unfeasible with a one-mode approach, although material incompatibilities between modes may be difficult in practice. Another new trend is composite printing for new functions, such as hybrid microfluidic/electronic systems [102].

Fluid handling is a ubiquitous and tedious operation, such as cell culture media in the benchtop research and bodily fluids in clinical diagnostics. Usually, fluids are transferred between containers by pipettors (prone to operator’s error) or expensive robotic dispensers. Pumps, valves, and mixers are critical for the fluids manipulation and automation to reduce labor costs, speed up processing, and enable mass parallelization. PDMS valves always outperform plastic valves of similar size. The invention of PDMS micro-valves and pumps revolutionizes the microfluidics and heralds the miniaturization and automation of multiple biomedical assays. The printed valves can also work as functional modules. For example, two valves in pair act as a switch while three valves in series as a peristaltic pump. The absence of standardization in interfacing PDMS devices with the peripherals is a major bottleneck in the widespread adoption of LOC technologies since the inlet/outlet connectors and tubing are usually the most unreliable components in the channels. SL-printed plastic 3D circuits with packaged connectors can be built as interlocking modules that represent existing industrial standards and are easy to operate. The introduction of modular design paradigms and integration of fluidic devices will amplify the efforts of individual teams for industrial success. “Plug and play” complex 3D milli-fluidic devices using flow control, inter-connectable modular devices, and both passive and active components allow the mixing, monitoring of reaction and cell culture progress [103]. A sample library of standardized components and connectors with validated flow characteristics has been established, which would allow the design and assemble complex 3D microfluidic circuit as easily as that in the electronics industry [100]. In addition, the modular design can also allow the access to interior surfaces of microchannels to improve the optical transparency using either mechanical or chemical processes [104] so that analysis on these resulting chips could be more accurate and reliable although transparent 3D printing materials now have limited availability. Fluidic devices with active valves and pumps as small as 10% of the volume and up to 1 million actuation could be manufactured by DLP-SL and used for serial multiplexer and mixer [105]. User-friendly fluid automation devices in transparent and biocompatible channels can be printed by non-engineers and integrated with other microfluidic devices as the replacement for costly robotic pipettors or tedious manual pipetting. Printing these devices requires the digital file of various modules for new device assembly and reconstruction with expanded functionality as well as recyclability and electronic access to a printer by non-expert users without facility limitations. The combination of rubber O-rings and metal pins improve module connectivity. The inserted O-rings perfectly seal the interconnections between the modules firmly and prevent leakage.

Because the predicted performance of a complex multi-layer PDMS device from the ideal design is usually different from the real performance, drastically reducing the number of fabrication iterations in the development of a complex device will save time and resources significantly. The ability to fabricate a complex microfluidic device in a single step has obvious advantages but challenges [106]. Despite the enthusiasm of the early uptakers, its applicability is limited partially by the technical inability to print microfluidic channels reliably with dimensions less than several hundred microns. MakerBot has created a very vibrant website (“Thingiverse”) for sharing some CAD designs with non-commercial (Creative Commons) licenses. In addition, 3DSkema will soon launch an online marketplace where designers can sell their licensed designs. Web-based 3D–printing services are becoming popular for designers owing to no requirement of expensive molds and “minimum quantity” limit in the production [107].

Currently, photopolymer resins are used in 3D printing technologies to make fluidic devices. New resins exhibiting improved optical transparency, gas permeability, and biocompatibility are continuously available, which will favor further applications of 3D printing in fluidic-based biological systems with optical measurement. Optical transparency allows on-chip detection while electrical insulation allows electrophoretic separation. In addition, thermoplastics and elastomers are used in non-photocurable techniques while soft hydrogels in bioprinters. Cell viability is the key parameter in the cytotoxicity tests of chemicals, cellular stress assays, DNA sorting, single-cell behavior and cell manipulation, especially organ-on-a-chip devices. The biocompatibility and bio-functionality of the available 3D printing materials are serious concerns but would facilitate cell attachment on the printed surface [25]. Free radical is a common concern associated with photo-polymerizable hydrogel in tissue engineering, where photo-initiator concentration compromises cell viability. Some of the most promising resins from a biomedical perspective (e.g., PEG-DA) are inexpensive and patent-free as they have been used as biomaterials (photo-cross-linkable hydrogels) for cell encapsulation for a long time. Although initial biosafety and biocompatibility studies of 3D–printed devices are encouraging (even implantable and bioresorbable), longer-term in vitro cytotoxicity and in vivo implant compatibility studies are greatly required. Zebrafish embryos cultured in 3D–printed structures made of Visijet crystal or Watershed showed developmental defects, but no behavioral abnormalities were found among those grown in leachate extracts of ABS and PLA.

Gradient generators, droplet extractors, and isotachophoresis chips are successfully generated for the future low-cost analytical applications. The ability to extract, purify, label, or separate the sample within the device helps to reduce analysis time and improve throughput. Samples are then sensed and detected using optical (e.g., fluorescence), electrochemical (e.g., conductivity, amperometry, and potentiometry), mass spectrometry, or biosensors. New microfluidic designs integrating electrodes and membrane inserts are successfully employed in the electrochemical detection of neurotransmitters and viruses, the collection of biologically relevant analysts (e.g., ATP), and drug transportation [108].

Humidity, variance in the gelatin viscosity, dispenser condition, and the printing parameters (e.g., air pressure, valve opening time, and droplet spacing) can also influence the channel diameter and structures. Air pressure and valve opening time mainly influence the channel width whereas the sequential number of printing has a higher impact on the channel height. Increased channel width results in the decreased migration speed of the cells so that cancer cells move faster in smaller veins than in large arteries. In addition, the proliferation of HUVECs is suppressed under the flow condition, which corresponds with previous studies that a long-term exposure of the endothelium to shear inhibits cell proliferation and reduces the metabolic rate [109]. Although culture media were consistently supplied, there was a limitation on maintaining cell viability of vascular channel probably because of high oxygen/nutrient consumption when a large number of cells was embedded nearby the channel. In addition, the cell viability decreases at the higher concentration of gelatin encapsulation or the long incubation time for gelatin liquefaction. One of the future directions of 3D bio-printing is to create implantable thick vascularized tissue constructs that could serve as artificial organs or aid in their repair and regeneration.

Advances in 3D cell printing technology have enabled the direct assembly of cells and extracellular matrix materials to form in vitro cellular models for biology (e.g., hypoxia, tissue repair), the evolution of disease pathogenesis and new drug discovery in printed 3D tumor models in vitro [110]. Construction of a multi-layered transport/μ-vascular channel network in a range of 100–300 μm has a resolution of tens of microns. Although the most effective way in tumor studies and anti-cancer drug screening is in clinical trials, ethical and safety limitations prevent it from wide acceptance. To overcome this hurdle, preclinical tumor models are used to mimic physiological tumorgenesis environments [111]. However, immunocompromised mice in use may show false effects on tumor development and progression [112]. Constructing 3D microstructures can provide a virtual environment that mimics the physical condition in vivo appropriate for the growth of cells or micro-organisms to a large extent and allow experiments to be conducted with a more clinical or biological relevance compared to culture in a Petri dish or flask [113]. Using this technology, the composition of vascular cells and supporting cells, flow rate/flow pattern, injection of soluble factors/small molecules, and other media components could be altered easily. Thus, vascular channels can be served as an experimental model for diverse vascular disease-related studies, for example, inflammation, immune responses, and tumor angiogenesis [85]. The lack of a simple and effective method to integrate vascular network with engineered scaffolds and tissue constructs is one of the greatest challenges in 3D tissue engineering currently. Translation of 2D fabrication methods into a 3D complex vascularized tissue construct, integration of channels across layers in the dimension of millimeters and its mechanical stability should be paid attention [56]. Neural stem cells can also be embedded along with vasculatures and growth factors to examine their mutual effect on network formation. The maturation process of the engineered vasculature, capillary formation, and angiogenic sprouting provide insight into the EC behavior under 3D flow conditions for the investigation of vascular biology. 3D cell printing has been reported in the printing of in vitro liver tissues [114], adipose tissues [115], bone tissues [116] and hybrid tissue constructs with vascular-like networks [117].

Technical challenges in this field include, but are not limited to the requirement of increased resolution, speed, and compatibility with biologically relevant materials [35]. The speed of fabrication should be increased for constructs in clinically relevant sizes. Rapid improvements in 3D printing resolution, even in low-cost consumer-grade desktop 3D printers, are highly possible for the rapid prototyping and cost-effective fabrication in high resolution and therefore more precise control of fluid flow [93]. The ability to image, map, and reproduce complex 3D structures composed of biologically relevant ECM proteins would be a major advancement for the applications. Microenvironment for long-term cell culture and growth in a user-friendly, highly-controllable, and broadly-accessible manner would advance the applicability of 3D printing to engineering physiological systems [118]. The viability of encapsulated cells is impacted by the processing time of the pre-gel bio-ink preparation and the construct deposition and the sensitivity of different cell types to external stresses. The use of multi-nozzle printheads (e.g., photopolymer, UV, microplasma, bioink, etc) [119] for co-printing cell-laden hydrogels could decrease the printing time and maximize the cell viability [120, 121]. It can be dedicated to printing the tissue constructs and the microfluidic channels simultaneously. The intersection of 3D printing for microfluidic fabrication and bioprinting 3D tissues shows great promise to organ-on-a-chip in single-step. With a combinatorial single-step fabrication, organ-on-a-chip would be far more accessible and cost-effective due to faster design iterations and shorter turnaround times. Instrumented cardiac microphysiological devices via multimaterial 3D printing has been fabricated to monitor drug responses and non-invasively measure tissue contractile stresses inside human stem cell-derived laminar cardiac cell via embedded sensors over 4 weeks [122]. Cell reprogramming and directed differentiation may provide high proliferation, functionality, nonimmunogenity and robust cell populations [35]. Combination of various mature and multipotent cells can be applied to efficiently reproduce the cell phenotypes for specific tissue targets. Small embryoid bodies (EBs) are more likely to have cardiomyocyte differentiation toward ectoderm while larger EBs towards endoderm and mesoderm. Spontaneous aggregation of inducible stem cells and EBs leads to the inhomogeneous size distribution of EBs and unpredictability in lineage differentiation. Thus, control of EBs in uniform sizes and shapes is beneficial in tissue engineering and regenerations [123]. 3D bioprinter can be further integrated with minimally invasive surgical robots to improve the healing procedure of the tissues removed by the surgical intervention. With the use of induced pluripotent stem cells derived from patients and differentiated into particular cell types, it will eventually be possible to generate organs-on-a-chip with a patient’s own cells as personalized medical treatment. Furthermore, a body-on-a-chip with multiple organs organized on a single chip to better model the multiorgan interactions in vivo is one of the investigative directions.

Simulation coupled with experiment can help in understanding the effects of printing parameters on cell viability [91]. Simulation can predict cell fate and provide more parametric control over 3D cancer models as well as complex viable tissue surrogates. A finite-difference/front-tracking simulation model was presented for deposition of viscous compound droplets onto a receiving surface with the inclusion of significant hydrodynamic pressures, capillary forces, and shear stresses. Several parameters, such as Weber number, diameter ratio, viscosity ratio, Reynolds number, surface tension ratio, and equilibrium contact angle were investigated for their influences on the transient deformation of a double emulsion droplet during bio-printing. Such strategy can accelerate the incorporation of 3D bioprinting technologies into cancer research and the development of more precise and reliable anticancer drug delivery systems.

## Conclusions

Microfluidic channel combined with 3D printing is emerging as a powerful tool in lab-on-a-chip and biological study. With the fast technical development in the design complexity, manufacture resolution and throughput, more applications will bring the technology to wide acceptance and commercialization. Overall, the development needs the integration of multidisciplinary technologies in engineering, biomaterials, cell biology, physics, and medicine [35].
